# Correction: The efficacy of novel biomarkers for the early detection and management of acute kidney injury: A systematic review

**DOI:** 10.1371/journal.pone.0330280

**Published:** 2025-08-12

**Authors:** Mohammed Yousef Almulhim

The Funding statement for this article is incorrect. The correct Funding statement is as follows: This research was funded by the Deanship of Scientific Research at King Faisal University, Saudi Arabia (GRANT A487).
