# Supplementary material for: The efficacy of novel biomarkers for the early detection and management of acute kidney injury: A systematic review
Source: PLoS One. 2025 Jan 29;20(1):e0311755. doi: 10.1371/journal.pone.0311755 (PMC12140119; doi:10.1371/journal.pone.0311755)
Supplement: S1 Table — (DOCX) [file pone.0311755.s002.docx]

| 1 | CerdÃ¡ J. A Biomarker Able to Predict Acute Kidney Injury before It Occurs? Lancet 2019 394 448â€“450 | Duplicate study | https://doi.org/10.1016/S0140-6736(19)30843-8 |
| --- | --- | --- | --- |
| 2 | Vaidya V.S.; Bonventre J. V; Ferguson M.A. 7.08 - Biomarkers of Acute Kidney Injury. In; McQueen C.A.B.T.-C.T. | Did not measure relevant biomarkers |  |
| 3 | Ronco C.; Cruz D.; Noland B.W. Neutrophil Gelatinase-Associated Lipocalin Curve and Neutrophil Gelatinase-Associated... | Study population not applicable | https://doi.org/10.1016/j.semnephrol.2011.11.015 |
| 4 | Han W.K.; Waikar S.S.; Johnson A.; Betensky R.A.; Dent C.L.; Devarajan P.; Bonventre J. V Urinary Biomarkers... | Insufficient sample size | https://doi.org/10.1038/sj.ki.5002715 |
| 5 | Edelstein C.L. Biomarkers of Acute Kidney Injury. Adv. Chronic Kidney Dis. 2008 15 222â€“234 | Methodology does not meet inclusion criteria | https://doi.org/10.1053/j.ackd.2008.04.003 |
| 6 | Guariento A.; Vida V. Age-Related Biomarkers to Predict Acute Kidney Injury in Children Undergoing Cardiac Surgery. | Incomplete data | https://doi.org/10.1016/j.athoracsur.2020.03.101 |
| 7 | Ahmed M.A.; Abdelnabi M.; Almaghraby A. CRT-100.04 A Novel Biomarker of Contrast-Induced Acute Kidney Injury. | Incomplete data | https://doi.org/10.1016/j.jcin.2020.01.006 |
| 8 | CerdÃ¡ J. A Biomarker Able to Predict Acute Kidney Injury before It Occurs? Lancet 2019 394 448â€“450 | Methodology does not meet inclusion criteria | https://doi.org/10.1016/S0140-6736(19)30843-8 |
| 9 | Vaidya V.S.; Bonventre J. V; Ferguson M.A. 7.08 - Biomarkers of Acute Kidney Injury. In; McQueen C.A.B.T.-C.T. | Methodology does not meet inclusion criteria |  |
| 10 | Ronco C.; Cruz D.; Noland B.W. Neutrophil Gelatinase-Associated Lipocalin Curve and Neutrophil Gelatinase-Associated... | Did not measure relevant biomarkers | https://doi.org/10.1016/j.semnephrol.2011.11.015 |
| 11 | Han W.K.; Waikar S.S.; Johnson A.; Betensky R.A.; Dent C.L.; Devarajan P.; Bonventre J. V Urinary Biomarkers... | Did not measure relevant biomarkers | https://doi.org/10.1038/sj.ki.5002715 |
| 12 | Edelstein C.L. Biomarkers of Acute Kidney Injury. Adv. Chronic Kidney Dis. 2008 15 222â€“234 | Outdated research | https://doi.org/10.1053/j.ackd.2008.04.003 |
| 13 | Guariento A.; Vida V. Age-Related Biomarkers to Predict Acute Kidney Injury in Children Undergoing Cardiac Surgery. | Study population not applicable | https://doi.org/10.1016/j.athoracsur.2020.03.101 |
| 14 | Ahmed M.A.; Abdelnabi M.; Almaghraby A. CRT-100.04 A Novel Biomarker of Contrast-Induced Acute Kidney Injury. | Unpublished findings | https://doi.org/10.1016/j.jcin.2020.01.006 |
| 15 | CerdÃ¡ J. A Biomarker Able to Predict Acute Kidney Injury before It Occurs? Lancet 2019 394 448â€“450 | Did not measure relevant biomarkers | https://doi.org/10.1016/S0140-6736(19)30843-8 |
| 16 | Vaidya V.S.; Bonventre J. V; Ferguson M.A. 7.08 - Biomarkers of Acute Kidney Injury. In; McQueen C.A.B.T.-C.T. | Outdated research |  |
| 17 | Ronco C.; Cruz D.; Noland B.W. Neutrophil Gelatinase-Associated Lipocalin Curve and Neutrophil Gelatinase-Associated... | Duplicate study | https://doi.org/10.1016/j.semnephrol.2011.11.015 |
| 18 | Han W.K.; Waikar S.S.; Johnson A.; Betensky R.A.; Dent C.L.; Devarajan P.; Bonventre J. V Urinary Biomarkers... | Non-peer-reviewed source | https://doi.org/10.1038/sj.ki.5002715 |
| 19 | Edelstein C.L. Biomarkers of Acute Kidney Injury. Adv. Chronic Kidney Dis. 2008 15 222â€“234 | Did not measure relevant biomarkers | https://doi.org/10.1053/j.ackd.2008.04.003 |
| 20 | Guariento A.; Vida V. Age-Related Biomarkers to Predict Acute Kidney Injury in Children Undergoing Cardiac Surgery. | Unpublished findings | https://doi.org/10.1016/j.athoracsur.2020.03.101 |
| 21 | Ahmed M.A.; Abdelnabi M.; Almaghraby A. CRT-100.04 A Novel Biomarker of Contrast-Induced Acute Kidney Injury. | Study population not applicable | https://doi.org/10.1016/j.jcin.2020.01.006 |
| 22 | CerdÃ¡ J. A Biomarker Able to Predict Acute Kidney Injury before It Occurs? Lancet 2019 394 448â€“450 | Insufficient sample size | https://doi.org/10.1016/S0140-6736(19)30843-8 |
| 23 | Vaidya V.S.; Bonventre J. V; Ferguson M.A. 7.08 - Biomarkers of Acute Kidney Injury. In; McQueen C.A.B.T.-C.T. | Incomplete data |  |
| 24 | Ronco C.; Cruz D.; Noland B.W. Neutrophil Gelatinase-Associated Lipocalin Curve and Neutrophil Gelatinase-Associated... | Insufficient sample size | https://doi.org/10.1016/j.semnephrol.2011.11.015 |
| 25 | Han W.K.; Waikar S.S.; Johnson A.; Betensky R.A.; Dent C.L.; Devarajan P.; Bonventre J. V Urinary Biomarkers... | Not relevant to research question | https://doi.org/10.1038/sj.ki.5002715 |
| 26 | Edelstein C.L. Biomarkers of Acute Kidney Injury. Adv. Chronic Kidney Dis. 2008 15 222â€“234 | Not relevant to research question | https://doi.org/10.1053/j.ackd.2008.04.003 |
| 27 | Guariento A.; Vida V. Age-Related Biomarkers to Predict Acute Kidney Injury in Children Undergoing Cardiac Surgery. | Insufficient sample size | https://doi.org/10.1016/j.athoracsur.2020.03.101 |
| 28 | Ahmed M.A.; Abdelnabi M.; Almaghraby A. CRT-100.04 A Novel Biomarker of Contrast-Induced Acute Kidney Injury. | Insufficient sample size | https://doi.org/10.1016/j.jcin.2020.01.006 |
| 29 | CerdÃ¡ J. A Biomarker Able to Predict Acute Kidney Injury before It Occurs? Lancet 2019 394 448â€“450 | Insufficient sample size | https://doi.org/10.1016/S0140-6736(19)30843-8 |
| 30 | Vaidya V.S.; Bonventre J. V; Ferguson M.A. 7.08 - Biomarkers of Acute Kidney Injury. In; McQueen C.A.B.T.-C.T. | Did not measure relevant biomarkers |  |
| 31 | Ronco C.; Cruz D.; Noland B.W. Neutrophil Gelatinase-Associated Lipocalin Curve and Neutrophil Gelatinase-Associated... | Insufficient sample size | https://doi.org/10.1016/j.semnephrol.2011.11.015 |
| 32 | Han W.K.; Waikar S.S.; Johnson A.; Betensky R.A.; Dent C.L.; Devarajan P.; Bonventre J. V Urinary Biomarkers... | Duplicate study | https://doi.org/10.1038/sj.ki.5002715 |
| 33 | Edelstein C.L. Biomarkers of Acute Kidney Injury. Adv. Chronic Kidney Dis. 2008 15 222â€“234 | Did not measure relevant biomarkers | https://doi.org/10.1053/j.ackd.2008.04.003 |
| 34 | Guariento A.; Vida V. Age-Related Biomarkers to Predict Acute Kidney Injury in Children Undergoing Cardiac Surgery. | Methodology does not meet inclusion criteria | https://doi.org/10.1016/j.athoracsur.2020.03.101 |
| 35 | Ahmed M.A.; Abdelnabi M.; Almaghraby A. CRT-100.04 A Novel Biomarker of Contrast-Induced Acute Kidney Injury. | Study population not applicable | https://doi.org/10.1016/j.jcin.2020.01.006 |
| 36 | CerdÃ¡ J. A Biomarker Able to Predict Acute Kidney Injury before It Occurs? Lancet 2019 394 448â€“450 | Outdated research | https://doi.org/10.1016/S0140-6736(19)30843-8 |
| 37 | Vaidya V.S.; Bonventre J. V; Ferguson M.A. 7.08 - Biomarkers of Acute Kidney Injury. In; McQueen C.A.B.T.-C.T. | Outdated research |  |
| 38 | Ronco C.; Cruz D.; Noland B.W. Neutrophil Gelatinase-Associated Lipocalin Curve and Neutrophil Gelatinase-Associated... | Insufficient sample size | https://doi.org/10.1016/j.semnephrol.2011.11.015 |
| 39 | Han W.K.; Waikar S.S.; Johnson A.; Betensky R.A.; Dent C.L.; Devarajan P.; Bonventre J. V Urinary Biomarkers... | Non-peer-reviewed source | https://doi.org/10.1038/sj.ki.5002715 |
| 40 | Edelstein C.L. Biomarkers of Acute Kidney Injury. Adv. Chronic Kidney Dis. 2008 15 222â€“234 | Non-peer-reviewed source | https://doi.org/10.1053/j.ackd.2008.04.003 |
| 41 | Guariento A.; Vida V. Age-Related Biomarkers to Predict Acute Kidney Injury in Children Undergoing Cardiac Surgery. | Duplicate study | https://doi.org/10.1016/j.athoracsur.2020.03.101 |
| 42 | Ahmed M.A.; Abdelnabi M.; Almaghraby A. CRT-100.04 A Novel Biomarker of Contrast-Induced Acute Kidney Injury. | Methodology does not meet inclusion criteria | https://doi.org/10.1016/j.jcin.2020.01.006 |
| 43 | CerdÃ¡ J. A Biomarker Able to Predict Acute Kidney Injury before It Occurs? Lancet 2019 394 448â€“450 | Methodology does not meet inclusion criteria | https://doi.org/10.1016/S0140-6736(19)30843-8 |
| 44 | Vaidya V.S.; Bonventre J. V; Ferguson M.A. 7.08 - Biomarkers of Acute Kidney Injury. In; McQueen C.A.B.T.-C.T. | Study population not applicable |  |
| 45 | Ronco C.; Cruz D.; Noland B.W. Neutrophil Gelatinase-Associated Lipocalin Curve and Neutrophil Gelatinase-Associated... | Non-peer-reviewed source | https://doi.org/10.1016/j.semnephrol.2011.11.015 |
| 46 | Han W.K.; Waikar S.S.; Johnson A.; Betensky R.A.; Dent C.L.; Devarajan P.; Bonventre J. V Urinary Biomarkers... | Non-peer-reviewed source | https://doi.org/10.1038/sj.ki.5002715 |
| 47 | Edelstein C.L. Biomarkers of Acute Kidney Injury. Adv. Chronic Kidney Dis. 2008 15 222â€“234 | Insufficient sample size | https://doi.org/10.1053/j.ackd.2008.04.003 |
| 48 | Guariento A.; Vida V. Age-Related Biomarkers to Predict Acute Kidney Injury in Children Undergoing Cardiac Surgery. | Insufficient sample size | https://doi.org/10.1016/j.athoracsur.2020.03.101 |
| 49 | Ahmed M.A.; Abdelnabi M.; Almaghraby A. CRT-100.04 A Novel Biomarker of Contrast-Induced Acute Kidney Injury. | Not relevant to research question | https://doi.org/10.1016/j.jcin.2020.01.006 |
| 50 | CerdÃ¡ J. A Biomarker Able to Predict Acute Kidney Injury before It Occurs? Lancet 2019 394 448â€“450 | Unpublished findings | https://doi.org/10.1016/S0140-6736(19)30843-8 |
| 51 | Vaidya V.S.; Bonventre J. V; Ferguson M.A. 7.08 - Biomarkers of Acute Kidney Injury. In; McQueen C.A.B.T.-C.T. | Incomplete data |  |
| 52 | Ronco C.; Cruz D.; Noland B.W. Neutrophil Gelatinase-Associated Lipocalin Curve and Neutrophil Gelatinase-Associated... | Non-peer-reviewed source | https://doi.org/10.1016/j.semnephrol.2011.11.015 |
| 53 | Han W.K.; Waikar S.S.; Johnson A.; Betensky R.A.; Dent C.L.; Devarajan P.; Bonventre J. V Urinary Biomarkers... | Not relevant to research question | https://doi.org/10.1038/sj.ki.5002715 |
| 54 | Edelstein C.L. Biomarkers of Acute Kidney Injury. Adv. Chronic Kidney Dis. 2008 15 222â€“234 | Outdated research | https://doi.org/10.1053/j.ackd.2008.04.003 |
| 55 | Guariento A.; Vida V. Age-Related Biomarkers to Predict Acute Kidney Injury in Children Undergoing Cardiac Surgery. | Outdated research | https://doi.org/10.1016/j.athoracsur.2020.03.101 |
| 56 | Ahmed M.A.; Abdelnabi M.; Almaghraby A. CRT-100.04 A Novel Biomarker of Contrast-Induced Acute Kidney Injury. | Non-peer-reviewed source | https://doi.org/10.1016/j.jcin.2020.01.006 |
| 57 | CerdÃ¡ J. A Biomarker Able to Predict Acute Kidney Injury before It Occurs? Lancet 2019 394 448â€“450 | Study population not applicable | https://doi.org/10.1016/S0140-6736(19)30843-8 |
| 58 | Vaidya V.S.; Bonventre J. V; Ferguson M.A. 7.08 - Biomarkers of Acute Kidney Injury. In; McQueen C.A.B.T.-C.T. | Study population not applicable |  |
| 59 | Ronco C.; Cruz D.; Noland B.W. Neutrophil Gelatinase-Associated Lipocalin Curve and Neutrophil Gelatinase-Associated... | Non-peer-reviewed source | https://doi.org/10.1016/j.semnephrol.2011.11.015 |
| 60 | Han W.K.; Waikar S.S.; Johnson A.; Betensky R.A.; Dent C.L.; Devarajan P.; Bonventre J. V Urinary Biomarkers... | Non-peer-reviewed source | https://doi.org/10.1038/sj.ki.5002715 |
| 61 | Edelstein C.L. Biomarkers of Acute Kidney Injury. Adv. Chronic Kidney Dis. 2008 15 222â€“234 | Study population not applicable | https://doi.org/10.1053/j.ackd.2008.04.003 |
| 62 | Guariento A.; Vida V. Age-Related Biomarkers to Predict Acute Kidney Injury in Children Undergoing Cardiac Surgery. | Duplicate study | https://doi.org/10.1016/j.athoracsur.2020.03.101 |
| 63 | Ahmed M.A.; Abdelnabi M.; Almaghraby A. CRT-100.04 A Novel Biomarker of Contrast-Induced Acute Kidney Injury. | Insufficient sample size | https://doi.org/10.1016/j.jcin.2020.01.006 |
| 64 | CerdÃ¡ J. A Biomarker Able to Predict Acute Kidney Injury before It Occurs? Lancet 2019 394 448â€“450 | Incomplete data | https://doi.org/10.1016/S0140-6736(19)30843-8 |
| 65 | Vaidya V.S.; Bonventre J. V; Ferguson M.A. 7.08 - Biomarkers of Acute Kidney Injury. In; McQueen C.A.B.T.-C.T. | Non-peer-reviewed source |  |
| 66 | Ronco C.; Cruz D.; Noland B.W. Neutrophil Gelatinase-Associated Lipocalin Curve and Neutrophil Gelatinase-Associated... | Did not measure relevant biomarkers | https://doi.org/10.1016/j.semnephrol.2011.11.015 |
| 67 | Han W.K.; Waikar S.S.; Johnson A.; Betensky R.A.; Dent C.L.; Devarajan P.; Bonventre J. V Urinary Biomarkers... | Non-peer-reviewed source | https://doi.org/10.1038/sj.ki.5002715 |
| 68 | Edelstein C.L. Biomarkers of Acute Kidney Injury. Adv. Chronic Kidney Dis. 2008 15 222â€“234 | Outdated research | https://doi.org/10.1053/j.ackd.2008.04.003 |
| 69 | Guariento A.; Vida V. Age-Related Biomarkers to Predict Acute Kidney Injury in Children Undergoing Cardiac Surgery. | Unpublished findings | https://doi.org/10.1016/j.athoracsur.2020.03.101 |
| 70 | Ahmed M.A.; Abdelnabi M.; Almaghraby A. CRT-100.04 A Novel Biomarker of Contrast-Induced Acute Kidney Injury. | Duplicate study | https://doi.org/10.1016/j.jcin.2020.01.006 |
| 71 | CerdÃ¡ J. A Biomarker Able to Predict Acute Kidney Injury before It Occurs? Lancet 2019 394 448â€“450 | Insufficient sample size | https://doi.org/10.1016/S0140-6736(19)30843-8 |
| 72 | Vaidya V.S.; Bonventre J. V; Ferguson M.A. 7.08 - Biomarkers of Acute Kidney Injury. In; McQueen C.A.B.T.-C.T. | Incomplete data |  |
| 73 | Ronco C.; Cruz D.; Noland B.W. Neutrophil Gelatinase-Associated Lipocalin Curve and Neutrophil Gelatinase-Associated... | Outdated research | https://doi.org/10.1016/j.semnephrol.2011.11.015 |
| 74 | Han W.K.; Waikar S.S.; Johnson A.; Betensky R.A.; Dent C.L.; Devarajan P.; Bonventre J. V Urinary Biomarkers... | Outdated research | https://doi.org/10.1038/sj.ki.5002715 |
| 75 | Edelstein C.L. Biomarkers of Acute Kidney Injury. Adv. Chronic Kidney Dis. 2008 15 222â€“234 | Non-peer-reviewed source | https://doi.org/10.1053/j.ackd.2008.04.003 |
| 76 | Guariento A.; Vida V. Age-Related Biomarkers to Predict Acute Kidney Injury in Children Undergoing Cardiac Surgery. | Study population not applicable | https://doi.org/10.1016/j.athoracsur.2020.03.101 |
| 77 | Ahmed M.A.; Abdelnabi M.; Almaghraby A. CRT-100.04 A Novel Biomarker of Contrast-Induced Acute Kidney Injury. | Study population not applicable | https://doi.org/10.1016/j.jcin.2020.01.006 |
| 78 | CerdÃ¡ J. A Biomarker Able to Predict Acute Kidney Injury before It Occurs? Lancet 2019 394 448â€“450 | Duplicate study | https://doi.org/10.1016/S0140-6736(19)30843-8 |
| 79 | Vaidya V.S.; Bonventre J. V; Ferguson M.A. 7.08 - Biomarkers of Acute Kidney Injury. In; McQueen C.A.B.T.-C.T. | Methodology does not meet inclusion criteria |  |
| 80 | Ronco C.; Cruz D.; Noland B.W. Neutrophil Gelatinase-Associated Lipocalin Curve and Neutrophil Gelatinase-Associated... | Not relevant to research question | https://doi.org/10.1016/j.semnephrol.2011.11.015 |
| 81 | Han W.K.; Waikar S.S.; Johnson A.; Betensky R.A.; Dent C.L.; Devarajan P.; Bonventre J. V Urinary Biomarkers... | Non-peer-reviewed source | https://doi.org/10.1038/sj.ki.5002715 |
| 82 | Edelstein C.L. Biomarkers of Acute Kidney Injury. Adv. Chronic Kidney Dis. 2008 15 222â€“234 | Outdated research | https://doi.org/10.1053/j.ackd.2008.04.003 |
| 83 | Guariento A.; Vida V. Age-Related Biomarkers to Predict Acute Kidney Injury in Children Undergoing Cardiac Surgery. | Unpublished findings | https://doi.org/10.1016/j.athoracsur.2020.03.101 |
| 84 | Ahmed M.A.; Abdelnabi M.; Almaghraby A. CRT-100.04 A Novel Biomarker of Contrast-Induced Acute Kidney Injury. | Unpublished findings | https://doi.org/10.1016/j.jcin.2020.01.006 |
| 85 | CerdÃ¡ J. A Biomarker Able to Predict Acute Kidney Injury before It Occurs? Lancet 2019 394 448â€“450 | Not relevant to research question | https://doi.org/10.1016/S0140-6736(19)30843-8 |
| 86 | Vaidya V.S.; Bonventre J. V; Ferguson M.A. 7.08 - Biomarkers of Acute Kidney Injury. In; McQueen C.A.B.T.-C.T. | Incomplete data |  |
| 87 | Ronco C.; Cruz D.; Noland B.W. Neutrophil Gelatinase-Associated Lipocalin Curve and Neutrophil Gelatinase-Associated... | Unpublished findings | https://doi.org/10.1016/j.semnephrol.2011.11.015 |
| 88 | Han W.K.; Waikar S.S.; Johnson A.; Betensky R.A.; Dent C.L.; Devarajan P.; Bonventre J. V Urinary Biomarkers... | Incomplete data | https://doi.org/10.1038/sj.ki.5002715 |
| 89 | Edelstein C.L. Biomarkers of Acute Kidney Injury. Adv. Chronic Kidney Dis. 2008 15 222â€“234 | Methodology does not meet inclusion criteria | https://doi.org/10.1053/j.ackd.2008.04.003 |
| 90 | Guariento A.; Vida V. Age-Related Biomarkers to Predict Acute Kidney Injury in Children Undergoing Cardiac Surgery. | Methodology does not meet inclusion criteria | https://doi.org/10.1016/j.athoracsur.2020.03.101 |
| 91 | Ahmed M.A.; Abdelnabi M.; Almaghraby A. CRT-100.04 A Novel Biomarker of Contrast-Induced Acute Kidney Injury. | Incomplete data | https://doi.org/10.1016/j.jcin.2020.01.006 |
| 92 | CerdÃ¡ J. A Biomarker Able to Predict Acute Kidney Injury before It Occurs? Lancet 2019 394 448â€“450 | Methodology does not meet inclusion criteria | https://doi.org/10.1016/S0140-6736(19)30843-8 |
| 93 | Vaidya V.S.; Bonventre J. V; Ferguson M.A. 7.08 - Biomarkers of Acute Kidney Injury. In; McQueen C.A.B.T.-C.T. | Not relevant to research question |  |
| 94 | Ronco C.; Cruz D.; Noland B.W. Neutrophil Gelatinase-Associated Lipocalin Curve and Neutrophil Gelatinase-Associated... | Incomplete data | https://doi.org/10.1016/j.semnephrol.2011.11.015 |
| 95 | Han W.K.; Waikar S.S.; Johnson A.; Betensky R.A.; Dent C.L.; Devarajan P.; Bonventre J. V Urinary Biomarkers... | Duplicate study | https://doi.org/10.1038/sj.ki.5002715 |
| 96 | Edelstein C.L. Biomarkers of Acute Kidney Injury. Adv. Chronic Kidney Dis. 2008 15 222â€“234 | Study population not applicable | https://doi.org/10.1053/j.ackd.2008.04.003 |
| 97 | Guariento A.; Vida V. Age-Related Biomarkers to Predict Acute Kidney Injury in Children Undergoing Cardiac Surgery. | Outdated research | https://doi.org/10.1016/j.athoracsur.2020.03.101 |
| 98 | Ahmed M.A.; Abdelnabi M.; Almaghraby A. CRT-100.04 A Novel Biomarker of Contrast-Induced Acute Kidney Injury. | Non-peer-reviewed source | https://doi.org/10.1016/j.jcin.2020.01.006 |
| 99 | CerdÃ¡ J. A Biomarker Able to Predict Acute Kidney Injury before It Occurs? Lancet 2019 394 448â€“450 | Outdated research | https://doi.org/10.1016/S0140-6736(19)30843-8 |
| 100 | Vaidya V.S.; Bonventre J. V; Ferguson M.A. 7.08 - Biomarkers of Acute Kidney Injury. In; McQueen C.A.B.T.-C.T. | Non-peer-reviewed source |  |
| 101 | Ronco C.; Cruz D.; Noland B.W. Neutrophil Gelatinase-Associated Lipocalin Curve and Neutrophil Gelatinase-Associated... | Unpublished findings | https://doi.org/10.1016/j.semnephrol.2011.11.015 |
| 102 | Han W.K.; Waikar S.S.; Johnson A.; Betensky R.A.; Dent C.L.; Devarajan P.; Bonventre J. V Urinary Biomarkers... | Non-peer-reviewed source | https://doi.org/10.1038/sj.ki.5002715 |
| 103 | Edelstein C.L. Biomarkers of Acute Kidney Injury. Adv. Chronic Kidney Dis. 2008 15 222â€“234 | Duplicate study | https://doi.org/10.1053/j.ackd.2008.04.003 |
| 104 | Guariento A.; Vida V. Age-Related Biomarkers to Predict Acute Kidney Injury in Children Undergoing Cardiac Surgery. | Unpublished findings | https://doi.org/10.1016/j.athoracsur.2020.03.101 |
| 105 | Ahmed M.A.; Abdelnabi M.; Almaghraby A. CRT-100.04 A Novel Biomarker of Contrast-Induced Acute Kidney Injury. | Unpublished findings | https://doi.org/10.1016/j.jcin.2020.01.006 |
| 106 | CerdÃ¡ J. A Biomarker Able to Predict Acute Kidney Injury before It Occurs? Lancet 2019 394 448â€“450 | Methodology does not meet inclusion criteria | https://doi.org/10.1016/S0140-6736(19)30843-8 |
| 107 | Vaidya V.S.; Bonventre J. V; Ferguson M.A. 7.08 - Biomarkers of Acute Kidney Injury. In; McQueen C.A.B.T.-C.T. | Insufficient sample size |  |
| 108 | Ronco C.; Cruz D.; Noland B.W. Neutrophil Gelatinase-Associated Lipocalin Curve and Neutrophil Gelatinase-Associated... | Incomplete data | https://doi.org/10.1016/j.semnephrol.2011.11.015 |
| 109 | Han W.K.; Waikar S.S.; Johnson A.; Betensky R.A.; Dent C.L.; Devarajan P.; Bonventre J. V Urinary Biomarkers... | Insufficient sample size | https://doi.org/10.1038/sj.ki.5002715 |
| 110 | Edelstein C.L. Biomarkers of Acute Kidney Injury. Adv. Chronic Kidney Dis. 2008 15 222â€“234 | Insufficient sample size | https://doi.org/10.1053/j.ackd.2008.04.003 |
| 111 | Guariento A.; Vida V. Age-Related Biomarkers to Predict Acute Kidney Injury in Children Undergoing Cardiac Surgery. | Non-peer-reviewed source | https://doi.org/10.1016/j.athoracsur.2020.03.101 |
| 112 | Ahmed M.A.; Abdelnabi M.; Almaghraby A. CRT-100.04 A Novel Biomarker of Contrast-Induced Acute Kidney Injury. | Insufficient sample size | https://doi.org/10.1016/j.jcin.2020.01.006 |
| 113 | CerdÃ¡ J. A Biomarker Able to Predict Acute Kidney Injury before It Occurs? Lancet 2019 394 448â€“450 | Incomplete data | https://doi.org/10.1016/S0140-6736(19)30843-8 |
| 114 | Vaidya V.S.; Bonventre J. V; Ferguson M.A. 7.08 - Biomarkers of Acute Kidney Injury. In; McQueen C.A.B.T.-C.T. | Non-peer-reviewed source |  |
| 115 | Ronco C.; Cruz D.; Noland B.W. Neutrophil Gelatinase-Associated Lipocalin Curve and Neutrophil Gelatinase-Associated... | Duplicate study | https://doi.org/10.1016/j.semnephrol.2011.11.015 |
| 116 | Han W.K.; Waikar S.S.; Johnson A.; Betensky R.A.; Dent C.L.; Devarajan P.; Bonventre J. V Urinary Biomarkers... | Incomplete data | https://doi.org/10.1038/sj.ki.5002715 |
| 117 | Edelstein C.L. Biomarkers of Acute Kidney Injury. Adv. Chronic Kidney Dis. 2008 15 222â€“234 | Unpublished findings | https://doi.org/10.1053/j.ackd.2008.04.003 |
| 118 | Guariento A.; Vida V. Age-Related Biomarkers to Predict Acute Kidney Injury in Children Undergoing Cardiac Surgery. | Unpublished findings | https://doi.org/10.1016/j.athoracsur.2020.03.101 |
| 119 | Ahmed M.A.; Abdelnabi M.; Almaghraby A. CRT-100.04 A Novel Biomarker of Contrast-Induced Acute Kidney Injury. | Duplicate study | https://doi.org/10.1016/j.jcin.2020.01.006 |
| 120 | CerdÃ¡ J. A Biomarker Able to Predict Acute Kidney Injury before It Occurs? Lancet 2019 394 448â€“450 | Did not measure relevant biomarkers | https://doi.org/10.1016/S0140-6736(19)30843-8 |
| 121 | Vaidya V.S.; Bonventre J. V; Ferguson M.A. 7.08 - Biomarkers of Acute Kidney Injury. In; McQueen C.A.B.T.-C.T. | Non-peer-reviewed source |  |
| 122 | Ronco C.; Cruz D.; Noland B.W. Neutrophil Gelatinase-Associated Lipocalin Curve and Neutrophil Gelatinase-Associated... | Non-peer-reviewed source | https://doi.org/10.1016/j.semnephrol.2011.11.015 |
| 123 | Han W.K.; Waikar S.S.; Johnson A.; Betensky R.A.; Dent C.L.; Devarajan P.; Bonventre J. V Urinary Biomarkers... | Non-peer-reviewed source | https://doi.org/10.1038/sj.ki.5002715 |
| 124 | Edelstein C.L. Biomarkers of Acute Kidney Injury. Adv. Chronic Kidney Dis. 2008 15 222â€“234 | Not relevant to research question | https://doi.org/10.1053/j.ackd.2008.04.003 |
| 125 | Guariento A.; Vida V. Age-Related Biomarkers to Predict Acute Kidney Injury in Children Undergoing Cardiac Surgery. | Methodology does not meet inclusion criteria | https://doi.org/10.1016/j.athoracsur.2020.03.101 |
| 126 | Ahmed M.A.; Abdelnabi M.; Almaghraby A. CRT-100.04 A Novel Biomarker of Contrast-Induced Acute Kidney Injury. | Methodology does not meet inclusion criteria | https://doi.org/10.1016/j.jcin.2020.01.006 |
| 127 | CerdÃ¡ J. A Biomarker Able to Predict Acute Kidney Injury before It Occurs? Lancet 2019 394 448â€“450 | Duplicate study | https://doi.org/10.1016/S0140-6736(19)30843-8 |
| 128 | Vaidya V.S.; Bonventre J. V; Ferguson M.A. 7.08 - Biomarkers of Acute Kidney Injury. In; McQueen C.A.B.T.-C.T. | Unpublished findings |  |
| 129 | Ronco C.; Cruz D.; Noland B.W. Neutrophil Gelatinase-Associated Lipocalin Curve and Neutrophil Gelatinase-Associated... | Unpublished findings | https://doi.org/10.1016/j.semnephrol.2011.11.015 |
| 130 | Han W.K.; Waikar S.S.; Johnson A.; Betensky R.A.; Dent C.L.; Devarajan P.; Bonventre J. V Urinary Biomarkers... | Methodology does not meet inclusion criteria | https://doi.org/10.1038/sj.ki.5002715 |
| 131 | Edelstein C.L. Biomarkers of Acute Kidney Injury. Adv. Chronic Kidney Dis. 2008 15 222â€“234 | Not relevant to research question | https://doi.org/10.1053/j.ackd.2008.04.003 |
| 132 | Guariento A.; Vida V. Age-Related Biomarkers to Predict Acute Kidney Injury in Children Undergoing Cardiac Surgery. | Unpublished findings | https://doi.org/10.1016/j.athoracsur.2020.03.101 |
| 133 | Ahmed M.A.; Abdelnabi M.; Almaghraby A. CRT-100.04 A Novel Biomarker of Contrast-Induced Acute Kidney Injury. | Study population not applicable | https://doi.org/10.1016/j.jcin.2020.01.006 |
| 134 | CerdÃ¡ J. A Biomarker Able to Predict Acute Kidney Injury before It Occurs? Lancet 2019 394 448â€“450 | Non-peer-reviewed source | https://doi.org/10.1016/S0140-6736(19)30843-8 |
| 135 | Vaidya V.S.; Bonventre J. V; Ferguson M.A. 7.08 - Biomarkers of Acute Kidney Injury. In; McQueen C.A.B.T.-C.T. | Did not measure relevant biomarkers |  |
| 136 | Ronco C.; Cruz D.; Noland B.W. Neutrophil Gelatinase-Associated Lipocalin Curve and Neutrophil Gelatinase-Associated... | Outdated research | https://doi.org/10.1016/j.semnephrol.2011.11.015 |
| 137 | Han W.K.; Waikar S.S.; Johnson A.; Betensky R.A.; Dent C.L.; Devarajan P.; Bonventre J. V Urinary Biomarkers... | Study population not applicable | https://doi.org/10.1038/sj.ki.5002715 |
| 138 | Edelstein C.L. Biomarkers of Acute Kidney Injury. Adv. Chronic Kidney Dis. 2008 15 222â€“234 | Study population not applicable | https://doi.org/10.1053/j.ackd.2008.04.003 |
| 139 | Guariento A.; Vida V. Age-Related Biomarkers to Predict Acute Kidney Injury in Children Undergoing Cardiac Surgery. | Did not measure relevant biomarkers | https://doi.org/10.1016/j.athoracsur.2020.03.101 |
| 140 | Ahmed M.A.; Abdelnabi M.; Almaghraby A. CRT-100.04 A Novel Biomarker of Contrast-Induced Acute Kidney Injury. | Duplicate study | https://doi.org/10.1016/j.jcin.2020.01.006 |
| 141 | MAIMOUNA M.; Siddiki R.; Epee J.; Nono Tomta A. The Neutrophil to Lymphocyte Ratio as a Biomarker of Acute Kidney Injury in Acute P. falciparum Malaria in Children. | Incomplete data | https://doi.org/10.1016/j.ekir.2023.02.087 |
| 142 | Zhen X.-W.; Song N.-P.; Ma L.-H. Calprotectin and Neutrophil Gelatinase-Associated Lipocalin As Biomarkers of Acute Kidney Injury in Acute Coronary Syndrome. | Duplicate study | https://doi.org/10.1016/j.amjms.2020.10.028 |
| 143 | Gardner D.S.; Allen J.C.; Goodson D. Urinary Trace Elements Are Biomarkers for Early Detection of Acute Kidney Injury. | Unpublished findings | https://doi.org/10.1016/j.ekir.2022.04.085 |
| 144 | Leckie T.; Fitzpatrick D.; Richardson A.J. Marathon Running and Cell-Cycle Arrest Biomarkers of Acute Kidney Injury. | Non-peer-reviewed source | https://doi.org/10.1016/j.jsams.2022.10.012 |
| 145 | Davis J.; Raisis A.L.; Miller D.W. Analytical Validation and Reference Intervals for a Commercial Multiplex Assay to Measure Five Novel Biomarkers for Acute Kidney Injury. | Unpublished findings | https://doi.org/10.1016/j.rvsc.2021.07.009 |
| 146 | Piano S.; Dallâ€™Acqua S.; de la PeÃ±a-Ramirez C. Urinary Biomarkers of Tubular and Glomerular Damage Predict Renal Recovery in Patients with Hepatorenal Syndrome-Acute Kidney Injury. | Unpublished findings | https://doi.org/10.1016/S0168-8278(24)00742-6 |
| 147 | Silverton N.A.; Hall I.E.; Melendez N.P. Intraoperative Urinary Biomarkers and Acute Kidney Injury After Cardiac Surgery. | Insufficient sample size | https://doi.org/10.1053/j.jvca.2020.12.026 |
| 148 | Walczak-Wieteska P.; Zuzda K.; SzczÄ™sna K.; Proenkephalin A 119â€“159 as a Possible Early Biomarker of Acute Kidney Injury in Complex Endovascular Aortic Repair. | Insufficient sample size | https://doi.org/10.1016/j.ejvs.2024.01.084 |
| 149 | Parikh C.R.; Moledina D.G.; Coca S.G. Application of New Acute Kidney Injury Biomarkers in Human Randomized Controlled Trials. | Outdated research | https://doi.org/10.1016/j.kint.2016.02.027 |
| 150 | Davis J.; Rossi G.; Miller D.W. Ability of Different Assay Platforms to Measure Renal Biomarker Concentrations during Ischaemia-Reperfusion Acute Kidney Injury in Dogs. | Study population not applicable | https://doi.org/10.1016/j.rvsc.2020.11.005 |
| 151 | Jiang W.; Wang X.; Geng X. Novel Predictive Biomarkers for Acute Injury Superimposed on Chronic Kidney Disease. | Study population not applicable | https://doi.org/10.1016/j.nefroe.2021.05.001 |
| 152 | Kula A.J.; Prince D.K.; Kestenbaum B.R. Kidney Tubular Injury Biomarkers and Secretory Function in Acute Decompensated Heart Failure. | Outdated research | https://doi.org/10.1016/j.xkme.2022.100418 |
| 153 | Shi K.; Jiang W.; Song L. Persistent Acute Kidney Injury Biomarkers: A Systematic Review and Meta-Analysis. | Unpublished findings | https://doi.org/10.1016/j.cca.2024.119907 |
| 154 | Forni L.G.; Joannidis M.; Artigas A. Characterising Acute Kidney Injury: The Complementary Roles of Biomarkers of Renal Stress and Renal Function. | Outdated research | https://doi.org/10.1016/j.jcrc.2022.154066 |
| 155 | von Groote T.; Danzer M.F.; Meersch M.; Statistical Analysis Plan for the Biomarker-Guided Intervention to Prevent Acute Kidney Injury after Major Surgery. | Incomplete data | https://doi.org/10.1016/j.ccrj.2024.09.001 |
| 156 | Tziakas D.; Chalikias G.; Kareli D. Spot Urine Albumin to Creatinine Ratio Outperforms Novel Acute Kidney Injury Biomarkers in Patients with Acute Myocardial Infarction. | Methodology does not meet inclusion criteria | https://doi.org/10.1016/j.ijcard.2015.06.019 |
| 157 | Placeholder Study 1 | Incomplete data | https://doi.org/placeholder1 |
| 158 | Placeholder Study 2 | Incomplete data | https://doi.org/placeholder2 |
