# Supplementary material for: The efficacy of novel biomarkers for the early detection and management of acute kidney injury: A systematic review
Source: PLoS One. 2025 Jan 29;20(1):e0311755. doi: 10.1371/journal.pone.0311755 (PMC12140119; doi:10.1371/journal.pone.0311755)
Supplement: S1 Data — (DOCX) [file pone.0311755.s003.docx]

**Table 1: Studies Identified in the Literature Search**

| **Study ID** | **Title of Study** | **Included/Excluded** | **Reason for Exclusion (if applicable)** |
| --- | --- | --- | --- |
| Ruilope et ‎al.‎ | Efficacy and Safety of Esaxerenone (CS-3150) for the Treatment of Type 2 Diabetes with Microalbuminuria | Included |  |
| Haitao ‎Zhang et ‎al.‎ | Blood Pressure and Cardiorenal Outcomes With Finerenone in Chronic Kidney Disease and Type 2 Diabetes | Included |  |
| Filippatos ‎et al.‎ | Safety and Tolerability of BAY 94-8862 in Patients with Chronic Heart Failure | Included |  |
| Bakris et ‎al.‎ | Effect of KBP-5074 on Blood Pressure in Advanced Chronic Kidney Disease: Results of the BLOCK-CKD Study | Included |  |
| Sadayoshi Ito et al.‎ | Balcinrenone plus Dapagliflozin in Patients with Heart Failure and Chronic Kidney Disease: Results from the Phase 2b MIRACLE Trial | Excluded | Early trial termination due to slow recruitment |
| Pitt et al.‎ | Finerenone in Patients with Chronic Kidney Disease and Type 2 Diabetes: The FIDELIO-DKD Subgroup from China | Included |  |
| Carolyn ‎S.P. Lam ‎et al.‎ | Finerenone and Cardiovascular Outcomes in Patients with Chronic Kidney Disease and Type 2 Diabetes | Included |  |
| George ‎Bakris, et ‎al.‎ | Mineralocorticoid Receptor Antagonists for Hypertension Management in Advanced Chronic Kidney Disease | Included |  |
| George L. ‎Bakris(b) ‎et al.‎ | Design and Baseline Characteristics of the Finerenone in Reducing Cardiovascular Mortality and Morbidity in Diabetic Kidney Disease Trial | Included |  |
| Luis M. ‎Ruilope et ‎al.‎ | The 12-week MIRACLE Study on Balcinrenone plus Dapagliflozin in Patients with Heart Failure and CKD | Excluded | Premature termination before statistical power was achieved |

**Table 2: Extracted Data from Primary Studies**

| **Study ID** | **Title of Study** | **Data Extractor(s)** | **Date of Data Extraction** | **Confirmation of Eligibility** | **Key Data Extracted** |
| --- | --- | --- | --- | --- | --- |
| 1 | Efficacy and Safety of Esaxerenone (CS-3150) for the Treatment of Type 2 Diabetes with Microalbuminuria​(efficacy_and_safety_of_…) | Mohammed Yousef Almulhim | January 15, 2024 | Eligible | Urinary Albumin-to-Creatinine Ratio, Safety Profile, Hyperkalemia Incidence |
| 2 | Blood Pressure and Cardiorenal Outcomes With Finerenone in Chronic Kidney Disease and Type 2 Diabetes​(RUILOP~1) | Mohammed Yousef Almulhim | January 15, 2024 | Eligible | Systolic Blood Pressure Reductions, Renal and Cardiovascular Outcomes |
| 3 | Safety and Tolerability of BAY 94-8862 in Patients with Chronic Heart Failure and Mild or Moderate Chronic Kidney Disease​(eht187) | Mohammed Yousef Almulhim | January 15, 2024 | Eligible | Potassium Levels, Hyperkalemia Incidence, Albuminuria, BNP Reduction, Renal Function Decline |
| 4 | Effect of KBP-5074 on Blood Pressure in Advanced Chronic Kidney Disease: Results of the BLOCK-CKD Study​(Effect_of_KBP-5074_on_B…) | Mohammed Yousef Almulhim | January 15, 2024 | Eligible | Blood Pressure Reduction, Albuminuria, Hyperkalemia Incidence |
| 5 | Finerenone in Patients with Chronic Kidney Disease and Type 2 Diabetes: The FIDELIO-DKD Subgroup from China​(000531997) | Mohammed Yousef Almulhim | January 15, 2024 | Eligible | Primary Composite Outcome: Kidney Failure, Decrease in eGFR, Renal Death, Secondary CV Outcomes |
| 6 | Finerenone and Cardiovascular Outcomes in Patients with Chronic Kidney Disease and Type 2 Diabetes​(cir-143-0540) | Mohammed Yousef Almulhim | January 15, 2024 | Eligible | Cardiovascular Death, Myocardial Infarction, Stroke, Heart Failure |
| 7 | Mineralocorticoid Receptor Antagonists for Hypertension Management in Advanced Chronic Kidney Disease​(BAKRIS~1) | Mohammed Yousef Almulhim | January 15, 2024 | Eligible | Systolic Blood Pressure, Renal Function, Hyperkalemia Incidence |
| 8 | Design and Baseline Characteristics of the Finerenone in Reducing Cardiovascular Mortality and Morbidity in Diabetic Kidney Disease Trial​(000503712) | Mohammed Yousef Almulhim | January 15, 2024 | Eligible | Study Design, Baseline Characteristics, Renal and Cardiovascular Outcomes |
| 9 | Balcinrenone plus Dapagliflozin in Patients with Heart Failure and Chronic Kidney Disease: Results from the Phase 2b MIRACLE Trial​(EUROPE~1) | Mohammed Yousef Almulhim | January 15, 2024 | Excluded | Early trial termination due to slow recruitment |
| 10 | Effect of Finerenone on Albuminuria in Patients with Diabetic Nephropathy: A Randomized Clinical Trial​(jama.2015.10081) | Mohammed Yousef Almulhim | January 15, 2024 | Eligible | Urinary Albumin-to-Creatinine Ratio, Safety Outcomes, Hyperkalemia Incidence |
