# Supplementary material for: The efficacy of novel biomarkers for the early detection and management of acute kidney injury: A systematic review
Source: PLoS One. 2025 Jan 29;20(1):e0311755. doi: 10.1371/journal.pone.0311755 (PMC12140119; doi:10.1371/journal.pone.0311755)
Supplement: Attachment — Submitted filename: A rebuttal letter.docx [file pone.0311755.s004.docx]

Dear Editor,

We thank the reviewers for their thorough evaluation of our manuscript. We appreciate the constructive comments and suggestions, which have helped us improve the clarity and quality of our work. Below, we provide detailed responses to each comment, along with the corresponding revisions made to the manuscript.

**Reviewer #1 Comments and Responses:**

**Comment 1:** While the manuscript effectively synthesizes findings from various studies, there are instances where the evidence presented lacks consistency in terms of study design, sample size, and methodology. It's crucial to ensure that the evidence supporting each claim is robust and coherent across different studies.

**Response:** We agree with this observation and have revised the manuscript to ensure consistency in the presentation of evidence. We have added a paragraph to the Discussion section that addresses the variability in study design, sample size, and methodology, and discusses how these factors may impact the findings.

**Comment 2:** The manuscript frequently discusses associations between biomarker levels and clinical outcomes (e.g., mortality, need for renal replacement therapy), but it's important to clarify whether these biomarkers directly contribute to these outcomes or merely serve as indicators of underlying renal injury. Clarifying the distinction between causality and association would strengthen the argument for the clinical relevance of these biomarkers.

**Response:** We agree that distinguishing between causality and association is crucial. We have revised the manuscript to clearly state when biomarkers are being used as indicators of underlying renal injury and when there is evidence to suggest a direct contribution to clinical outcomes. This includes a detailed discussion on the nature of these relationships based on current evidence.

**Comment 3:** While the manuscript acknowledges the ongoing validation of biomarkers across diverse settings, it's essential to emphasize the limitations of extrapolating findings from specific study populations to broader clinical contexts. Discussing potential factors influencing biomarker performance in different patient populations (e.g., age, comorbidities, medication use) would enhance the discussion on generalizability.

**Response:** We have expanded the discussion on the generalizability of biomarker findings, highlighting the limitations of extrapolating results from specific study populations to broader clinical contexts. We discuss how factors such as age, comorbidities, and medication use may influence biomarker performance and the implications for clinical practice.

**Question 1:** Are there any common limitations or biases across these methodologies that could affect the reliability of the findings?

**Response:** Common limitations and biases across the studies include small sample sizes, single-center designs, and variability in patient populations. Additionally, there may be inconsistencies in the timing of biomarker measurements and a lack of standardized protocols. These factors can affect the reliability and generalizability of the findings. We have discussed these limitations in the revised manuscript.

**Question 2:** The manuscript highlights the importance of serial biomarker assessment in guiding treatment escalation or de-escalation for AKI patients. Could you discuss the evidence supporting the use of biomarker trends in clinical decision-making? Are there any standard protocols or guidelines for interpreting biomarker dynamics in AKI management, and if not, what are the implications for clinical practice?

**Response:** Serial biomarker assessment provides dynamic insights into the progression or resolution of AKI. Evidence from several studies supports the use of biomarker trends to guide treatment decisions, such as early initiation of renal replacement therapy or intensifying monitoring. However, there are currently no standardized protocols for interpreting biomarker dynamics. This lack of standardization presents challenges for clinical practice, highlighting the need for further research to establish guidelines. We have included this discussion in the revised manuscript.

**Question 3:** Considering the discussions on the superiority of novel biomarkers over traditional markers like serum creatinine, are there any potential confounding factors or biases that could influence the comparative efficacy assessment? How do researchers account for these factors in study design and data analysis?

**Response:** Potential confounding factors include differences in patient demographics, baseline kidney function, and comorbid conditions. Researchers account for these factors by using multivariable analysis, adjusting for known confounders, and employing randomized controlled trial designs when possible. Despite these efforts, residual confounding can still impact the findings. We have discussed these considerations in the revised manuscript.

**Question 4:** The manuscript suggests that future studies should evaluate whether biomarker-guided preventive strategies can reduce AKI burden and improve survival. What specific study designs or methodologies would be most suitable for investigating the long-term impact of biomarker-based interventions on patient outcomes? Additionally, what are the ethical considerations associated with implementing biomarker-guided preventive strategies in clinical practice, particularly regarding patient autonomy and resource allocation?

**Response:** Suitable study designs include large-scale randomized controlled trials with long-term follow-up, cohort studies, and pragmatic trials embedded in clinical practice. Ethical considerations include ensuring informed consent, respecting patient autonomy, and addressing potential inequalities in access to biomarker testing and interventions. Resource allocation must be carefully considered to avoid exacerbating disparities in healthcare. We have expanded on these points in the revised manuscript.

**Reviewer #2 Comments and Responses:**

**Comment 1:** The topic of this study is different from the research protocol that loaded to the PROSPERO database. It seems the research was narrowed in context to COVID-19 which is irrelevant to the manuscript. If there is a technical mistake, I would recommend editing the protocol. In addition, there is another author present in the database, but in the manuscript, the author is not present.

**Response:** We will immediately rectify this by updating the PROSPERO registration to accurately reflect the scope of our current study on novel biomarkers for AKI. regarding the second author is an assistant that was mentioned in the acknowledgment

**Comment 2:** It would be a good idea to add a table to summarize 17 studies according to study type, patients, biomarker efficacy, etc.

**Response:** We appreciate this suggestion. The Table is already submitted as supplementary file

**Comment 3:** Among the 17 studies included in the review, some of them were not mentioned enough in the paper. For instance, it would be nice to give brief information why the author included Dekkers’s study.

**Response:** We acknowledge the need for more detailed explanations. We have included brief descriptions of all 17 studies, with a focus on why each study was selected, including Dekkers’s study. This provides a clearer rationale for their inclusion and relevance to our review.

**Minor Remarks:**

**1.** In the author’s information, the title of the author is present which should not be. Also, the first letters of each word in the university name should be capitalized.

**Response:** We have revised the author information section to remove titles and ensure that the university name is correctly capitalized.

**2.** The sentence in line 85 is not clear, suggested to improve.

**Response:** We have revised the sentence on line 85 for better clarity and readability.

**3.** The information given in line 92 is not present in the indicated reference.

**Response:** We have reviewed and corrected the reference on line 92 to ensure accuracy and relevance.

**4.** In table 1, there is a typo: “tabel 1”.

**Response:** We have corrected the typo in Table 1 to “Table 1”.

**5.** In table 1, when a database search was made according to search terms, a considerably different number of items were found. The search may need to be rechecked.

**Response:** We have rechecked the database search terms and numbers to ensure accuracy and consistency across all searches.

**6.** In table 1, some of the search terms are not suitable for the indicated database. For example, in Embase, some parentheses are required for suitable places. For ease of reading, I would suggest making those terms suitable for databases.

**Response:** We have revised the search terms in Table 1 to ensure they are appropriately formatted for each database, enhancing the clarity and accuracy of our search strategy.

**7.** In figure 1, there is a typo: “PRIMISA.”

**Response:** We have corrected the typo in Figure 1 to “PRISMA”.

**8.** In line 252, it mentions about 18 studies, but 17 studies were included in the review.

**Response:** We have corrected the inconsistency and ensured that the number of studies mentioned is accurate throughout the manuscript.

**9.** In figure 2, there is inconsistency in authors’ names: some of them were written in full name and some were written in the last name. Besides, for ease of access to studies, adding reference numbers is suggested.

**Response:** the Figure was automatically generated by ROBVIS

**10.** In line 450, the following three sentences can be difficult to understand for readers. It would be better if it can be described more.

**Response:** We have revised and clarified the sentences on line 450 to ensure they are more understandable for readers.

**11.** References should be suitable for the journal’s reference style. Besides, lack of information or incorrect information is available (e.g., doi of ref1 is not correct).

**Response:** We have revised the references to conform to the journal’s reference style and corrected any inaccurate or missing information. some article has no DOI available and registered using PMID .

We hope that these revisions and responses satisfactorily address the reviewers' comments. We believe that these changes have significantly improved the quality of our manuscript. Thank you for your consideration.
