# Supplementary material for: The efficacy of novel biomarkers for the early detection and management of acute kidney injury: A systematic review
Source: PLoS One. 2025 Jan 29;20(1):e0311755. doi: 10.1371/journal.pone.0311755 (PMC12140119; doi:10.1371/journal.pone.0311755)
Supplement: Attachment — Submitted filename: plose rebuttal.docx [file pone.0311755.s005.docx]

**Dear Editor**

Thank you for forwarding the reviewers' comments regarding our manuscript titled **The Efficacy of Novel Biomarkers for The Early Detection and Management of Acute Kidney Injury: A Systematic Review**" submitted to PLOS ONE . We appreciate the reviewers' insightful feedback and have carefully considered their suggestions. Below, we address each point raised and detail the revisions we have made to the manuscript.

**Reviewer #2’s Comments:**

1. **Author Titles in PLOS ONE:**
   - **Response:** We apologize for the oversight and have removed all titles from the authors' names in the manuscript to comply with the PLOS ONE guidelines.
2. **PROSPERO Registration Update:**
   - **Response:** We appreciate the reviewer pointing out the discrepancy regarding the PROSPERO registration. We had already requested the modification in the protocol , we also ask if its needed to get new registration ?
3. **Typographical Errors and Search Terms:**
   - **Response:** We have corrected the typo "Tabel 1" to "Table 1" and reviewed all search terms for accuracy and relevance to the study’s focus. We apologize for the oversight and appreciate the reviewer’s attention to detail.

**Reviewer #3’s Comments:**

1. **Zarboch Study Inclusion:**
   - **Response:** We have revised the text to reflect the dual impact of the Zarboch study on both prevention and clinical management of AKI, which now appears in both relevant sections of the manuscript to provide clarity on its implications.
2. **Clarification on NGAL as a Protective Biomarker:**
   - **Response:** We have added a detailed explanation on the role of NGAL, supported by recent studies, to clarify its dual function as both a biomarker and a potential protective agent in the context of AKI.
3. **Association Between Biomarkers and Type of Renal Injury:**
   - **Response:** Additional information has been incorporated to discuss how different biomarkers relate to various types of renal injuries. This includes a nuanced discussion on the specificity of biomarkers like KIM-1 and L-FABP in detecting structural versus functional kidney injuries.
4. **Biomarkers Prior to Procedures:**
   - **Response:** We have clarified this point by explaining that biomarkers can indicate a predisposition to injury rather than current damage, thus providing an opportunity for preemptive interventions. This has been detailed with references to recent literature and existing consensus statements.

We believe these revisions have strengthened the manuscript and addressed the concerns raised by the reviewers. We appreciate the opportunity to revise our manuscript and hope that the changes meet the journal’s standards for publication.

Thank you for considering our work. We look forward to the possibility of our manuscript’s publication in PLOS ONE.

**Sincerely,**
